# Real–time observation of interfacial ions during electrocrystallization

**DOI:** 10.1038/s41598-017-01048-0

**Published:** 2017-04-20

**Authors:** Masashi Nakamura, Takahiro Banzai, Yuto Maehata, Osamu Endo, Hiroo Tajiri, Osami Sakata, Nagahiro Hoshi

**Affiliations:** 1 Department Applied Chemistry and Biotechnology, Graduate School of Engineering, Chiba University, Yayoi–cho 1–33, Inage–ku, Chiba 263–8522 Japan; 2 Department of Organic and Polymer Materials Chemistry, Faculty of Engineering, Tokyo University of Agriculture and Technology, Naka–cho 2–24–16, Koganei, Tokyo 184–8588 Japan; 3 Research and Utilization Division, Japan Synchrotron Radiation Research Institute/SPring–8, Kouto 1–1–1, Sayo, Sayo–gun, Hyogo 679–5198 Japan; 4 Synchrotron X–ray Station at SPring–8, National Institute for Materials Science, Kouto 1–1–1, Sayo–gun, Hyogo 679–5148 Japan

## Abstract

Understanding the electrocrystallization mechanisms of metal cations is of importance for many industrial and scientific fields. We have determined the transitional structures during underpotential deposition (upd) of various metal cations on Au(111) electrode using time–resolved surface X–ray diffraction and step–scan IR spectroscopy. At the initial stage of upd, a characteristic intensity transient appears in the time–resolved crystal truncation rod depending on metal cations. Metal cations with relatively high coordination energies of hydration water are deposited in two steps: first, the hydrated metal cations approached the surface and are metastably located at the outer Helmholtz plane, then they are deposited via the destruction of the hydration shell. However, Tl^+^ and Ag^+^, which have low hydration energy, are rapidly adsorbed on Au(111) electrode without any metastable states of dehydration. Therefore, the deposition rate is strongly related to the coordination energy of the hydration water. Metal cations strongly interacting with the counter coadsorbed anions such as Cu^2+^ in sulfuric acid causes the deposition rate to be slower because of the formation of complexes.

## Introduction

Deposition and dissolution of metal cations on metal surface are important consideration in industrial processes such as electrolytic refinement, corrosion, and plating. Well–designed electrocrystallization techniques are necessary for various fields: the nanofabrication of electrodes for the electronic devices and the surface modification of nanoparticles used in catalysts^1^. Core–shell catalysts modified by hetero–atoms can significantly enhance chemical reactions^2, 3^. Atomically controlled first or second layer deposition techniques are a major issue for the preparation of nano–catalysts^4^.

Under potential deposition (upd) of metal ions can control a well–defined adsorbed structure at the first layer. Although the adsorption state of upd layer is different from that of the bulk deposition, it can be available for catalyst preparation, and it is also useful for fundamental research as a model of the initial stage of cation adsorption^5, 6^. Detailed structures of various upd layers have been studied at the static electrode potential using scanning tunneling microscopy (STM) and surface X–ray diffraction (SXRD). The deposition potential and in–plane structure of a upd layer depend on the metal cation and its interaction with the surface^7, 8^. Most recently, many studies of upd phenomena and applications have been developed in various scientific areas^9–12^.

During the formation of upd layer, counter anion plays a role in the promotion or inhibition of the adsorption of metal cation. Moreover, solvents as well as additives will also affect approach of metal cation to the surface because metal cations pass in the diffuse layer and across the electrical double layer (EDL). Thus, these interfacial species significantly affect the upd mechanism. Therefore, real time observations in the wide region of interface are necessary for understanding the electrocrystallization process.

The EDL, in particular, governs the kinetics of adsorption and desorption because of the presence of localized ions and solvent layers. In the EDL, hydrated ions are located at the Helmholtz plane and solvents are highly oriented in a huge electric field by the layer structure of the charged species. In an electrochemical reaction, these layered structures are destroyed when a reaction species approaches the surface. Since metal cations are hydrated in solution, the dehydration process is also important during upd. Recent time–resolved SXRD (TRSXRD) measurements revealed that there is a delay in the approach of alkali metal cations to surface because the interfacial water causes a blocking effect^13^. Therefore, the kinetics of approaching step in the EDL is significantly different from that of migration in the bulk phase, and the adsorption rate depends on the hydration structure, ionic valence, and ionic size.

In this study, the initial upd processes of Tl^+^, Ag^+^, Cu^2+^, Zn^2+^, Cd^2+^, and Bi^3+^ on Au(111) were investigated by using TRSXRD and time–resolved infrared spectroscopy. SXRD is the best method to use for the structural determination of ionic species in the EDL, and it can also make detections with high time and spatial resolutions^14–18^. The dynamic structure of metal cations and counter anions in the EDL were captured at a time resolution of the order of μs to ms. This paper also discusses the adsorption rate of various metal cations and compares them with the coordination energy of hydrated water.

## Results

### TRSXRD of Cu deposition

**Figure 1:**
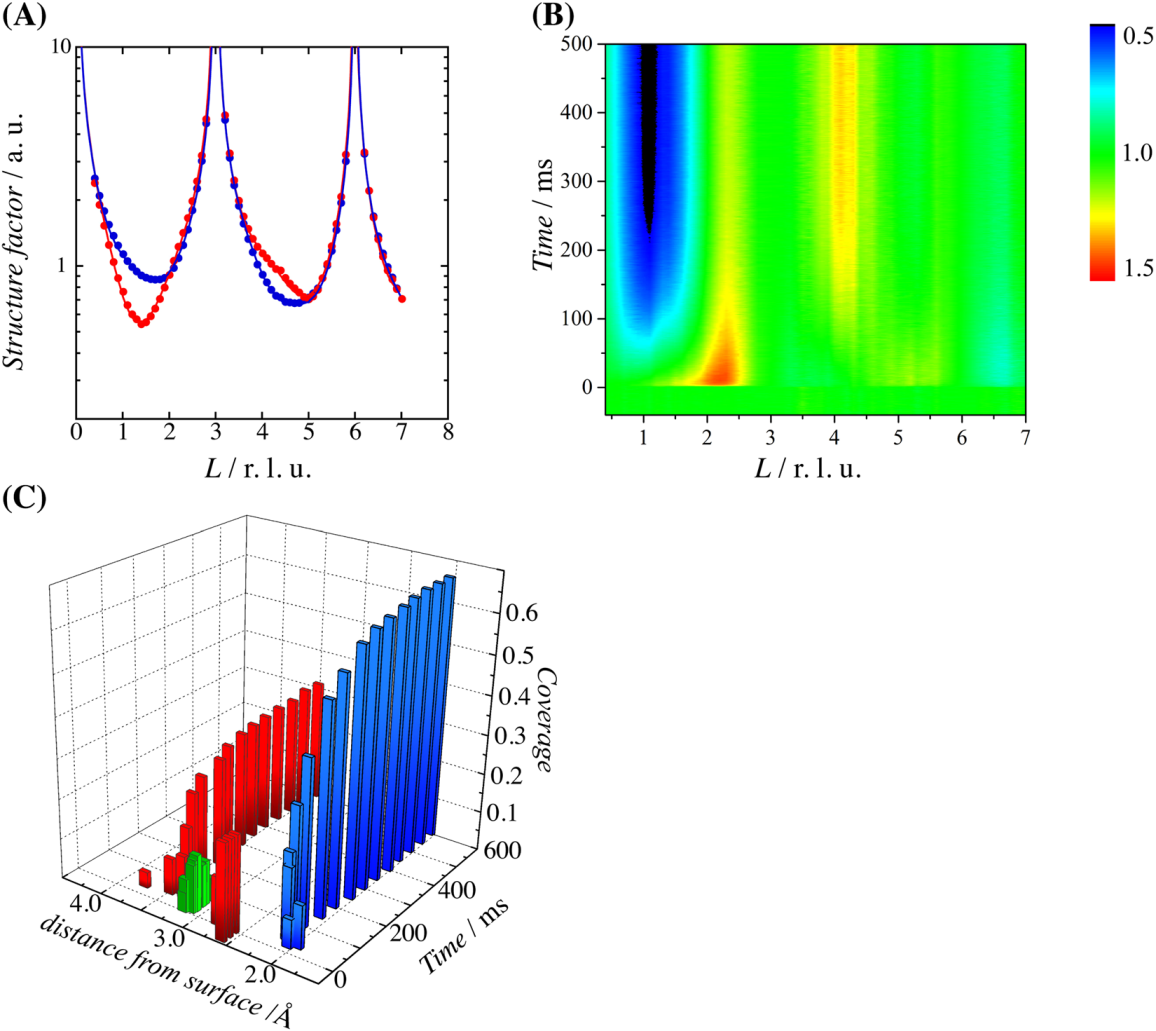
(**A**) Specular CTR of Au(111) in 0.5 M H_2_SO_4_ + 1.0 mM CuSO_4_ at the upd potential of 0.45 V (red points) and non–upd potential of 1.05 V vs SHE (blue points). The solid lines are the structure factors calculated from the optimized models. (**B**) Time–resolved specular CTR after the potential step from 1.05 V to 0.45 V with time resolution of 500 μs. The transient intensities were normalized against those at the non–upd potential of 1.05 V. Red and blue regions indicate the increase and decrease of intensity, respectively. (**C**) Time dependence of the structural parameters optimized using time–resolved specular CTR. The blue, red, and green bars represent the upd Cu, adsorbed (bi)sulfate, and non–adsorbed Cu, respectively. The position of adsorbed (bi)sulfate indicates the sulfur atom of tridentate (bi)sulfate.

Cu upd on Au(111) electrode in sulfuric acid occurs in two steps: the first is the upd of a honeycomb structure with a Cu coverage of 0.67, and the second step is that the Au(111) is fully covered by upd Cu with 1 × 1 structure^19–21^. The first upd is coadsorbed with a (bi)sulfate anion, which is located at the center of the honeycomb. In this study, the potentials step is applied from the non–upd potential of 1.05 V vs standard hydrogen electrode as a reference (SHE), forming well–ordered (bi)sulfate layer on Au(111) to the upd potential of 0.45 V, forming the first upd layer. Figure 1(A) shows the specular crystal truncation rod (CTR) profile of Au(111) in 0.5 M H_2_SO_4_ + 1.0 mM CuSO_4_ at 0.45 V and 1.05 V. The structural parameters in the surface normal direction were refined by the CTRs at 0.45 V and 1.05 V. The layer spacings and surface coverages of adsorbed species are consistent with those reported previously^20, 22, 23^.

We performed time–resolved measurements at peak positions along the CTR. Figure 1(B) shows the time–resolved intensity transient along the specular CTR after the step from 1.05 V to 0.45 V with time resolution of 500 μs. The intensities were normalized against those at the non–upd potential of 1.05 V. After 100 ms, the intensities around *L* = 1.2 decreases, whereas that around *L* = 4.2 increases. These changes can be ascribed to the deposition of the 0.67 ML Cu, according to the static potential results in Fig. 1(A). However, an abnormal intensity enhancement appears between *L* = 1.2 and 2.7 immediately after the potential step to 100 ms, and this cannot be attributed to the formation of the adsorbed layer. We constructed time–resolved structure factors along the specular CTR from the intensity transient in order to refine the structural parameters at each time. The structure factors are conventionally corrected from the integrated intensity measurement along the rod. We here assume that the integrated intensity is proportional to the peak intensity because the dynamic intensity change at the half maximum position is not caused by band broadening due to the structural inhomogeneity. The structure factor at each *L* position was estimated from the rate of change in the transient intensity based on the results of the static potentials^13^.

For the structural optimization, we first assume an initial model containing adsorbed sulfate and adsorbed upd Cu on Au(111). However, the increase of coverage of the adsorbed species reduces the CTR intensity at around *L* = 1.4. As the increase of the electronic density at the outer Helmholtz plane (OHP) often causes the enhancement in the CTR intensity between the Bragg points, depending on the distance from the surface^14–17^, we consider the outer layer species as well as the adsorbed species in the structural model. After the structural refinement, the initial enhancement after the step to the upd potential can be ascribed to the layer formation of the Cu species around 0.32 nm from the surface. When Cu is deposited on Au(111) surface directly, the distance between the first Au layer and the adsorbed Cu layer is 0.21 nm. Therefore, the distance of 0.32 nm indicates that this Cu species is not adsorbed directly but is located at the OHP as a hydrated state. During Cu deposition, hydrated Cu ions approach the OHP, and Cu ions are then adsorbed on Au(111) surface by the destruction of the hydration shell.

Figure 1(C) shows the time dependence of the structural parameters during the first upd of Cu. The adsorbed (bi)sulfate species at the non–upd potential are located at 0.27 nm from the Au(111) surface (Au–S layer spacing). After the potential is stepped to 0.45 V, (bi)sulfate species is desorbed within 1 ms, and then the hydrated Cu^2+^ immediately accumulates at the maximum coverage of 0.14. The coverage of the hydrated Cu^2+^ decreases after 40 ms, and the Cu and the (bi)sulfate are then adsorbed on Au(111) over the next 400 ms. The coverages of the adsorbed Cu and (bi)sulfate species correlates strongly, suggesting that these ions interact in a complementary manner.

### Time–resolved infrared spectroscopy of Cu deposition

XRD reflecting the electron density distribution cannot definitively distinguish the chemical species used. Therefore, we performed time–resolved infrared spectroscopy during Cu upd to reveal the time dependent structure of the adsorbed (bi)sulfate species. Conventional infrared reflection absorption spectroscopy (IRAS) using a single crystal electrode inhibits mass transfer in the interface because the surface is strongly pushed to the IR window. Surface enhanced infrared absorption spectroscopy (SEIRAS) with an attenuated total reflection (ATR) can achieve rapid mass transfer as is the case with the drop cell used in the TRSXRD. Although we cannot apply the well–defined single crystal electrode to the ATR–SEIRAS, we can use a quasi–Au(111) thin film on a Si prism prepared by thermal annealing^24^. The voltammetric features for the first and second upd on the quasi–Au(111) thin film are similar to those on the Au(111) single crystal electrode (Fig. S1).

**Figure 2:**
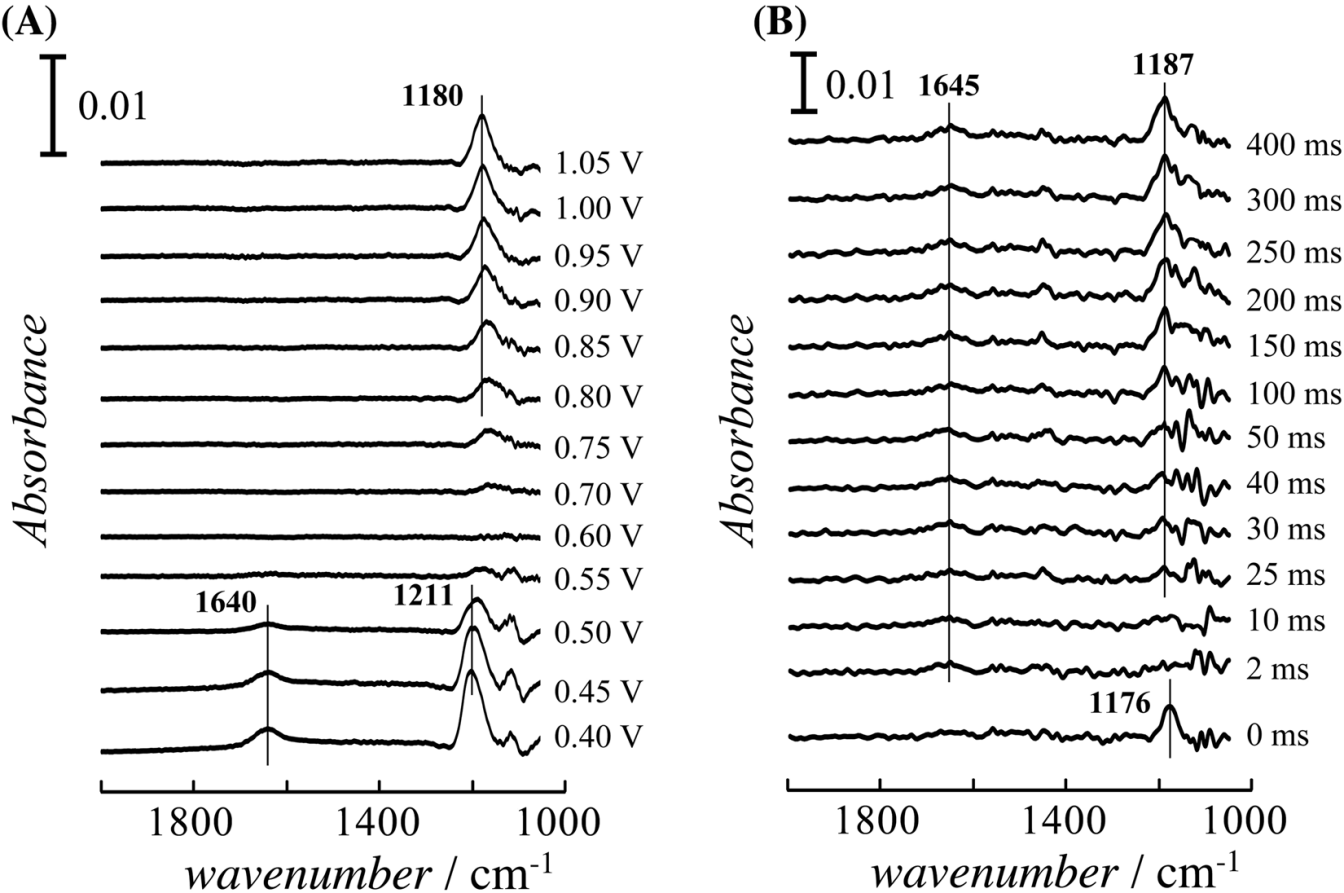
(**A**) Potential dependence of the SEIRAS of the quasi–Au(111) thin film on the Si prism in 0.5 M H_2_SO_4_ + 1.0 mM CuSO_4_. (**B**) TRSEIRAS obtained by the step–scan method after the potential step from 1.05 V to 0.45 V with time resolution of 500 μs. The reference spectra were corrected at 0.65 V vs SHE. The spectral resolutions of (**A**) and (**B**) were 4 cm^−1^ and 8 cm^−1^, respectively.

Figure 2(A) shows the potential dependence of the SEIRAS of the quasi–Au(111) thin film electrode in 0.5 M H_2_SO_4_ + 1 mM CuSO_4_; the band around 1200 cm^−1^ is assigned to the SO_3_ symmetric stretching mode (ν_SO3_) of tridentate (bi)sulfate^25, 26^. (Bi)sulfate anions are adsorbed on the bare Au surface above 0.75 V and coadsorbed with the upd Cu below 0.50 V, which is consistent with the spectra on Au(111) electrode using IRAS^27^. Figure 2(B) shows the time–resolved SEIRAS (TRSEIRAS), which were measured by the potential step from 1.05 V to 0.45 V. The spectral noise around 1100 cm^−1^ is due to the low transmittance of the Si prism. The low intensity band around 1450 cm^−1^, depends on the repetition frequency of the interferometer for time–resolved measurement, may be due to the external electric noise and vibration. The ν_SO3_ band observed at 0 ms disappears within 2 ms after the potential step to 0.45 V, and then its intensity gradually increases after 25 ms. The band intensity becomes constant after 300 ms. During the Cu deposition, the (bi)sulfate anion is initially desorbed from the Au surface, and then it is readsorbed by binding to the upd Cu. These results are consistent with the time–dependent coverage of the adsorbed (bi)sulfate obtained from the TRSXRD measurement, as shown in Fig. 1(C). The onset time of the Cu deposition corresponds with the adsorption of the (bi)sulfate anion, indicating that the deposition of Cu requires the coadsorbed (bi)sulfate to form an ionic complex.

The band at 1640 cm^−1^ observed in Cu upd region below 0.5 V may be assigned to the HOH bending mode (δ_HOH_) of the hydrogen bonded water layer covered on Cu upd as reported by a previous SXRD study^22^. The time–resolved spectra show that the band intensity of δ_HOH_ increases after the step to the upd potential. This result suggests that the orientation and surface coverage of interfacial water, including the Cu hydration shell, change after the desorption of the (bi)sulfate anion. However, a more precise assignment is difficult because the effective bond energies of these species are of the same order. The δ_HOH_ in the bulk phase also appears around 1640 cm^−1^.

In the previous IRAS and SEIRAS spectra of Fig. 2(A), the band frequencies of (bi)sulfate both on bare Au(111) and coadsorbed with Cu on Au(111) are 1180 and 1210 cm^−1^, respectively^27^. However, the band frequency after 25 ms is lower than that at the static potential of 0.45 V. The restitution of the frequency to 1210 cm^−1^ requires 10 s (not shown here), which is longer than the time needed to achieve the saturation coverage for the upd Cu and adsorbed sulfate. TRSXRD measurement was performed at (1/3 1/3 1.5), which is originated from the √3 × √3 honeycomb structure, as shown in Fig. S2. The convergence time needed is over 5 s. These results indicate that the in–plane rearrangement occurs to expand a well–ordered honeycomb domain after the Cu and sulfate achieve the saturation coverage. Previous STM study reported that the larger domains of the √3 × √3 grow at the expense of the small domain within a few minutes^28^.

### Comparison of other metal depositions with Cu upd

**Figure 3:**
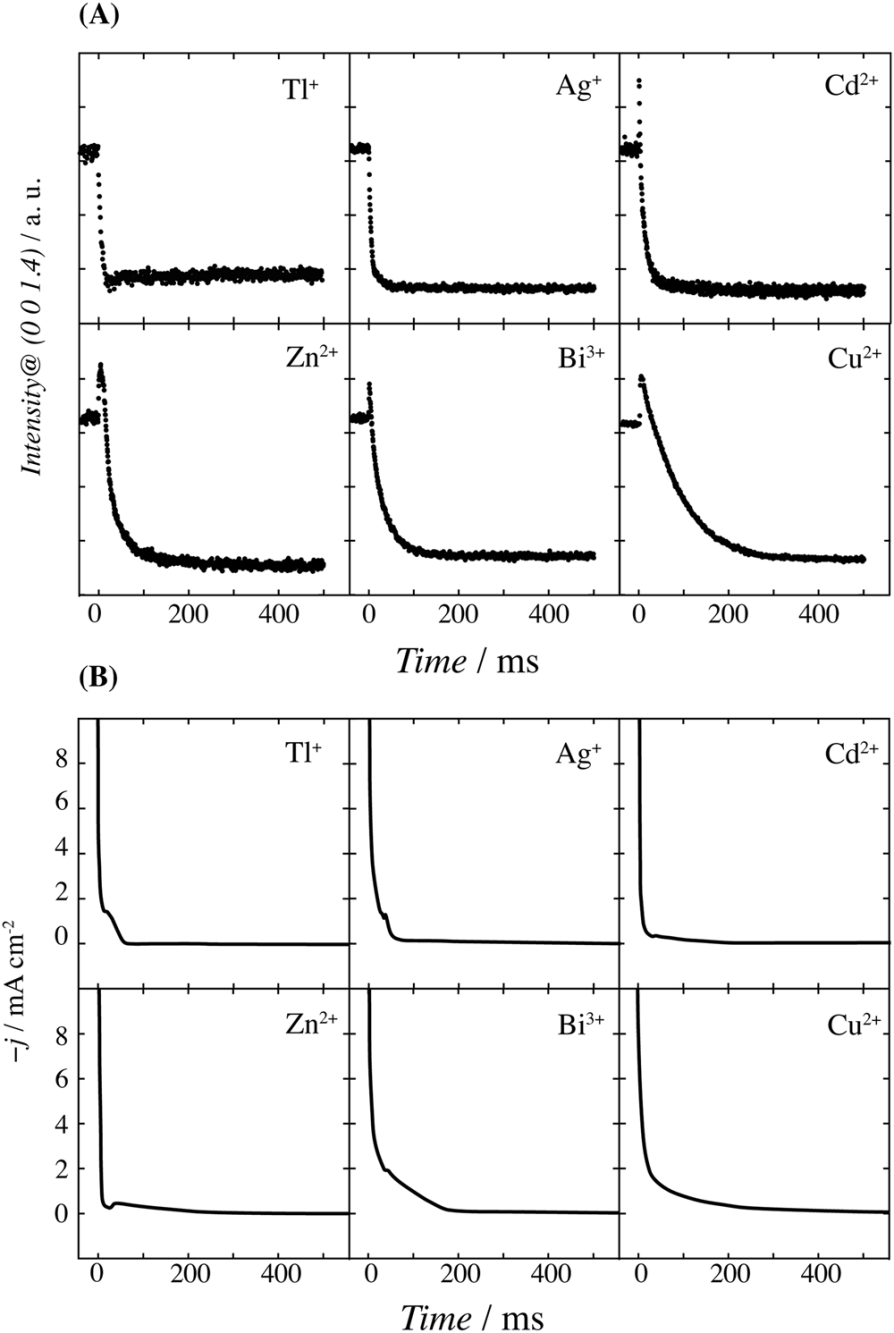
(**A**) TRSXRD at 0 0 1.4 with the time resolution of 500 μs and (**B**) current transient during the deposition of various metal cations on Au(111): Tl upd in 0.5 M H_2_SO_4_ + 0.5 mM Tl_2_SO_4_ form 0.20 V to −0.40 V, Ag upd in 0.5 M H_2_SO_4_ + 0.5 mM Ag_2_SO_4_ from 1.25 V to 0.75 V, Cd upd in 0.5 M H_2_SO_4_ + 1.0 mM CdSO_4_ from 0.60 V to 0.10 V, Zn upd in 0.5 M Na_2_HPO_4_ + 1.0 mM Zn(ClO_4_)_2_ from 0.30 V to −0.30 V, Bi upd in 1.0 M HClO_4_ + 0.5 mM Bi_2_O_3_ from 0.97 V to 0.37 V, Cu upd in 0.5 M H_2_SO_4_ + 1.0 mM CuSO_4_ from 1.05 V to 0.45 V.

The initial deposition processes were investigated for the upd of the different metal cations, and were compared with that of Cu upd. The potential of each upd layer was set to −0.40 V for Tl (0.67 ML), 0.75 V for Ag (1.0 ML), 0.1 V for Cd (0.5 ML), −0.3 V for Zn (0.33 ML), and 0.37 V for Bi (0.5 ML)^29–35^. Non–upd potentials were selected at significantly more positive (+0.5 ~ +0.6 V) than the upd potentials because the pre–cathodic current associated with upd often appears at more positive potential than the first upd peaks; examples of such peaks are in Cu upd and Bi upd (Fig. S3). If the non–upd and upd potentials are selected in each region with the same structural phase, the decay time of the intensity transient becomes independent from the upper and lower potentials. Figure 3(A) shows TRSXRD at the 0 0 1.4 reflection that is sensitive to each upd layer as shown in Fig. S4. The intensity transients of Cd, Zn, and Bi upd are similar to that of Cu upd; the intensity abruptly increases after the potential step before then decreasing exponentially. At the non–upd potentials for Cd, Zn, and Bi upd, no specifically adsorbed anions are adsorbed on Au(111); this supports the observation that the intensity increase is not because of the desorption of anions but rather because of the existence of temporal hydrated cation layer. Therefore, Cd^2+^, Zn^2+^, and Bi^3+^ are also deposited via a hydration state in a manner similar to that of Cu upd. However, Tl and Ag upd processes are different from the other upd processes; the intensity transients decrease without any temporary enhancement in intensity. The initial enhancement did not appear even at the faster time resolution of 100 μs. The intensity transients of other reciprocal lattice space also rapidly converge to the diffraction intensity of the upd potential, which indicates that Tl^+^ and Ag^+^ are deposited without a metastable state at the OHP.

The transient intensities *I* are well–fitted by the following exponential equation

$$I=k\exp (-\frac{t}{\tau })$$

where *k* and *τ* are the amplitude and time constant, respectively. The time constants were *τ*_Tl_ = 7.6 ms, *τ*_Ag_ = 9.6 ms, *τ*_Cd_ = 15.3 ms, *τ*_Zn_ = 24.9 ms, *τ*_Bi_ = 30.5 ms, and *τ*_Cu_ = 82.9 ms. The charge transfer from the electrode to cations is a key step in electrocrystallization. Intensity transients are compared with the current transients obtained using the same cell geometry with X–ray diffraction as shown in Fig. 3(B). The convergent times of each intensity transient correspond with those of the current transient. Since the charge transfer is far faster than migration and dehydration processes of ions, this suggests that the charge transfer occurs after or simultaneously with dehydration of metal cation. Although small or shoulder peaks assigned to the nucleation and growth processes depending on the metal appears in the current transient^36^, the amperometric features do not relate to the X–ray intensity transient. Current transient includes a variety of faradaic and non–faradaic processes during metal deposition, which do not necessarily correlate with the X–ray intensity that is sensitive to the electron density distribution.

## Discussion

**Figure 4:**
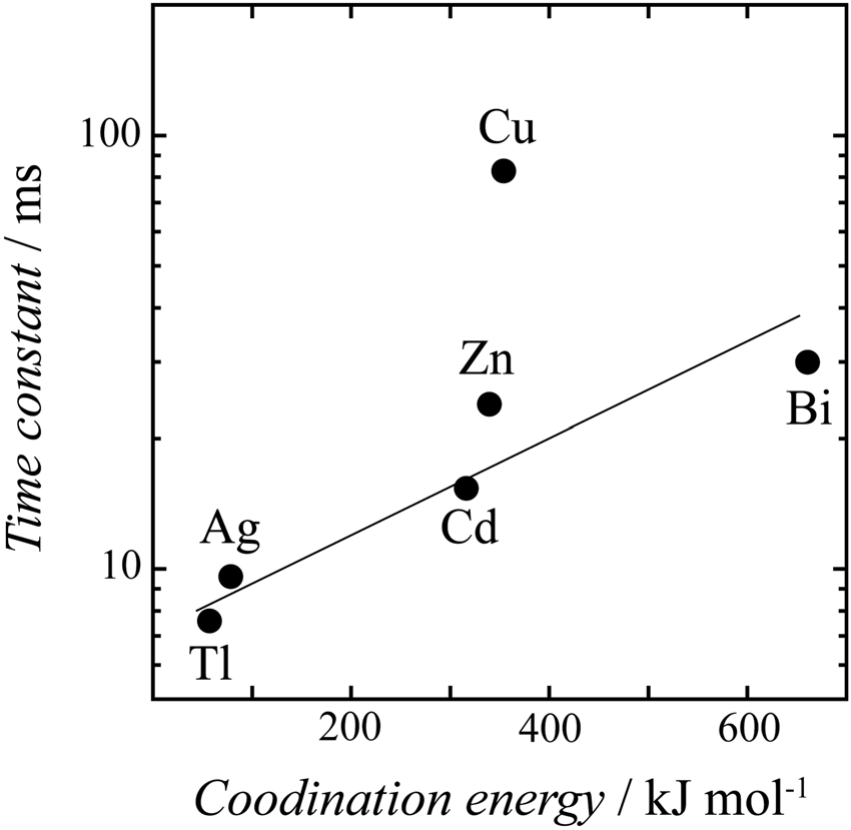
Correlation between the time constant of X–ray intensity decay and the coordination energy of each single hydration water of M(H_2_O)_6_^n+^.

As described above, except Tl and Ag upd, the metal cation with the hydration shell is metastably layered in the EDL before the deposition step. Therefore, the destruction of the hydration shell may be kinetically controlled step during deposition. We herein discuss the relation between the deposition rate and the coordination energy of the hydrated water. The hydration structure of metal cation at the OHP is different from that of the bulk phase because of the interaction with surface and the surrounding ions, which complicates the understanding of the dehydration process during upd. Therefore, hexa–aqua octahedral complex was used for simplification. The coordination energies of metal complexes, which consists of six hydrated water as the first hydration shell and the polarizable continuum model (PCM) as the outer hydration shell, are estimated using DFT calculations. Total coordination energies calculated using this model are consistent with the experimental and theoretical hydration enthalpies^37, 38^. Figure 4 shows the plot of the coordination energies against the time constants of the intensity transient. The coordination energy strongly relates to the ionic valence due to the electrostatic interaction. The deposition rate of the metal cations has a linear relationship with the coordination energy, except in the case of Cu upd; this fact indicates that strong interaction between water and the metal cation delays the adsorption to the surface. For any deposition processes on the electrode surface, interfacial water and preadsorbed anion will affect the adsorption of metal cation. However, the desorption of (bi)sulfate anion completes within 2 ms as described above and the migration and reorientation of interfacial water also occur within a few tens of μs – a few ms, according to previous time resolved studies^13, 39^. Thus, the time scale of the restructuring of preadsorbed species is faster than that of metal deposition investigated in this study.

**Figure 5:**
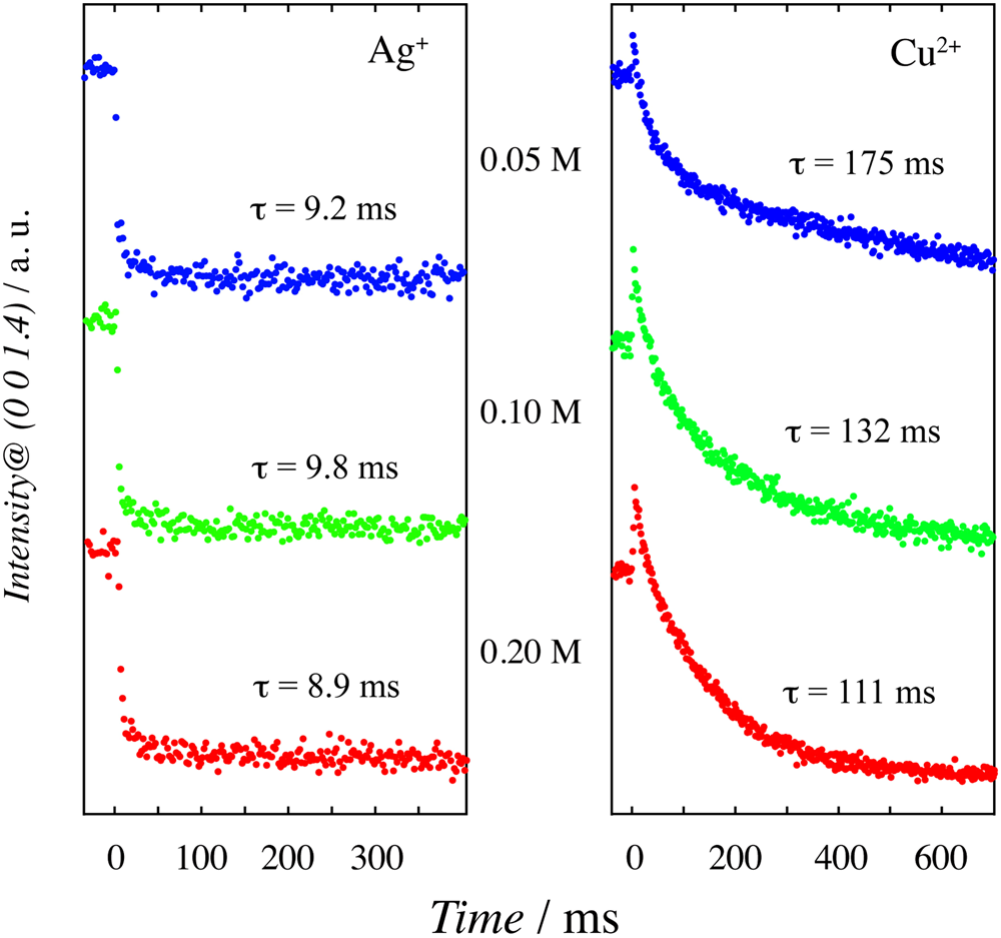
TRSXRD at 0 0 1.4 in *x* M H_2_SO_4_ + 0.5 mM Ag_2_SO_4_ and *x* M H_2_SO_4_ + 1.0 mM CuSO_4_ (*x* = 0.20 (red), 0.10 (green), and 0.05 (blue)) with the time resolution of 1.0 ms. The pH of all of the solutions is adjusted to be the same as that of 0.5 M H_2_SO_4_.

The decay time of the Cu deposition is slower than that of the Bi that a higher coordination energy. The slow time decay of Cu upd is due to its complex formation with the (bi)sulfate anion. The concentration dependence of the (bi)sulfate anion was investigated for the deposition rate of Cu and Ag upd to confirm the effect of the formation of the complex. Figure 5 shows the concentration dependence of TRSXRD on sulfuric acid for Ag and Cu upd. The solutions were adjusted to be the same pH by adding HClO_4_ to equalize the ratio between [SO_4_^2−^] and [HSO_4_^−^]. Although the decay of Ag upd is constant and did not depend on the concentration of sulfuric acid, the decay of Cu upd is faster as the concentration of sulfuric acid increases. In the case of Cu upd in 0.05 M H_2_SO_4_, the decay convergence does not occur even at 600 ms. It is known that the coadsorbed anions in the Cu upd layer strongly affect the onset potential of the metal deposition and the in–plane structure^40^. The absence, or reduced presence, of the (bi)sulfate anion that strongly interacts with Cu causes the peak broadening of the first upd in voltammogram^41^. This result supports that the deposition rate of Cu upd depends on [SO_4_^2−^] or [HSO_4_^−^]. As described above, the TRXRD and SEIRAS results show that the Cu and (bi)sulfate are adsorbed simultaneously, suggesting that there is strong affinity between the Cu^2+^ and sulfate anions. Since the potential of Cu upd is lower than that of the sulfate adsorption on Au(111) (i.e. negative potential than the pzc), the sulfate anions adjacent to the EDL should be less at the Cu upd potential. In the case of Bi upd, the perchlorate anion is not specifically adsorbed on the Bi upd layer. Therefore, the complex formation process of Cu requiring SO_4_ on Au(111) delays the Cu deposition and makes it slower than that of Bi upd.

In general, the counter anions affect the deposition processes of other upd cations as well as Cu. The sulfate and phosphate anions used in this study, are specifically adsorbed on the Ag, Cd, and Zn upd layers^32, 34, 42^. Although previous STM studies show that the electrolytes affect the in–plane structure of the Ag upd layer^43^, their electrochemical features in H_2_SO_4_, such as the onset potential and charge density, are quite similar to those in HClO_4_^44^. For Zn upd, the phosphate anions induce the Zn deposition forming Zn–phosphate complex^45^. However, the concentration of the phosphate anion does not affect the peak sharpness of the peak in voltammogram^45^, suggesting that the concentration of the counter anion does not affect the Zn deposition rate. These anions are directly adsorbed on the upd layers without strong complex formations. Thus, the destruction of the hydration shell and the affinity with the counter anion are important factors during the initial deposition process of metal cations.

In summary, the initial processes during metal deposition have been investigated on Au(111) using TRSXRD and IR measurements. In general, the adsorption rate of the metal cations is related to the coordination energy of the hydration water. However, for metal depositions that are accompanied by strong complex formation with the counter anion, the factor that determines the deposition rate is the concentration of the counter anion. Metal depositions with greater coordination energies of hydration water occurs in two steps: first, the hydrated metal cations approach the outer layer, and then the cations are deposited by the destruction of the hydration shell.

## Methods

An Au(111) disk crystal (MaTech, Julich, Germany) was used for SXRD and IR measurements. The sample was annealed in a H_2_ flame and then cooled to room temperature in an Ar atmosphere. The clean surface was protected with ultrapure water and transferred to an electrochemical cell. The drop cell for the electrochemical and X–ray measurements was used with a double junction type Hg/HgSO_4_ reference and the Au counter electrodes immersed in the electrolyte droplet on the surface. The potentials were referenced to that of the SHE. The concentration of the metal cations was prepared to be 1.0 mM.

SXRD measurements were performed with a multi–axis diffractometer at BL13XU (SPring–8) and BL4C (KEK PF). For the time–resolved experiment, a rectangular potential wave between non–upd and upd potentials was applied to the Au electrode with 1.0 and 0.2 Hz. Diffracted photons were detected by a Ce doped yttrium aluminum perovskite (YAP:Ce) detector, and the discriminated pulses were counted by a multichannel scaler (Ortec) synchronized with the function generator for potential control^13^. The diffraction intensity was accumulated over tens of thousands of cycles with a time resolution of either 500 μs or 1.0 ms. The potential response of the electrochemical system, depending on the configuration of the counter and reference electrodes, was less than 10 μs for the potential pulses used in this study. The X–ray beam energies were 15 and 20 keV. A hexagonal coordinate system was used to describe the reciprocal vector as *Q* = Ha* + Kb* + Lc*, where a* = b* = 2π/*a*, c* = √2π/*a*, *a* = 2.884 Å, and *L* is the direction normal to the surface. The structure refinement was performed using the least–squares method with the ANA–ROD program^46^.

SEIRAS was performed using a semi–cylindrical Si prism plated by an Au thin film^47^. Before the electroless deposition of Au, the surface was mechanically polished using alumina slurry. The Si oxide film on the prism was removed with 30% NH_4_F solution for 1 min. The plating solution (15 mM NaAuCl_4_ + 2% HF) was reduced on the Si prism by 150 mM Na_2_SO_3_ + 50 mM Na_2_S_2_O_3_ + 50 mM NH_4_Cl for 2 min at 60 °C. After rinsed with ultrapure water, the Au–coated Si prism was annealed to prepare the quasi–Au(111) surface.

IR light was incident through the Si prism at an angle of 60 °C. An IR electrochemical cell was attached to a Fourier–transform IR spectrometer (Bruker Vertex70v) with a photo voltaic mercury cadmium telluride (PV–MCT) detector. TRSEIRAS measurements were performed using the step scan method, and the potential step from non–upd to upd was applied at each interferogram point during the staircase step of the moving mirror. Transient interfering light was collected with the time resolution of 500 μs and the spectral resolution of 8 cm^−1^, and 10 interferograms were coadded at each time. The conventional measurement at the constant potential was performed with 4 cm^−1^ resolution and was averaged over 128 scans.

DFT calculations were performed with the Gaussian 03 program using the LANL2DZ ECP basis for the metal cations and the 6–311++G** basis for O and H at the B3LYP level. The coordination energy (Δ*E*_cood_) of the hexa–aqua complexes was estimated using the following equation:

$${\rm{\Delta }}{E}_{{\rm{cood}}}={E}_{{[{\rm{M}}{({{\rm{H}}}_{2}{\rm{O}})}_{6}]}^{n+}}-{E}_{{{\rm{M}}}^{n+}}-6{E}_{{{\rm{H}}}_{2}{\rm{O}}}$$

where ${E}_{{[{\rm{M}}{({{\rm{H}}}_{2}O)}_{6}]}^{n+}},{E}_{{{\rm{M}}}^{n+}}\,{\rm{and}}\,{E}_{{{\rm{H}}}_{2}{\rm{O}}}$ are the energies of [M(H_2_O)_6_]^n+^, M^n+^, and H_2_O, respectively. The PCM as solvent effect was applied for the energy calculations of [M(H_2_O)_6_]^n+^ and H_2_O.

## Electronic supplementary material

Supplimentary infomation
