# Supplementary material for: Real–time observation of interfacial ions during electrocrystallization
Source: Sci Rep. 2017 Apr 20;7:914. doi: 10.1038/s41598-017-01048-0 (PMC5430517; doi:10.1038/s41598-017-01048-0)
Supplement: Supplementary file 1 — Supplimentary infomation [file 41598_2017_1048_MOESM1_ESM.pdf]

Real−time observation of interfacial ions during the initial electroplating

Masashi Nakamura[1], Takahiro Banzai[1], Yuto Maehata[1], Osamu Endo[2], Hiroo Tajiri[3], Osami Sakata[4], Nagahiro Hoshi[1]

[1]Department Applied Chemistry and Biotechnology, Graduate School of Engineering, Chiba University, Yayoi−cho 1−33, Inage−ku, Chiba 263−8522, Japan.

[2]Department of Organic and Polymer Materials Chemistry, Faculty of Engineering, Tokyo University of Agriculture and Technology, Naka−cho 2−24−16, Koganei, Tokyo 184−8588, Japan.

[3]Research and Utilization Division, Japan Synchrotron Radiation Research Institute / SPring−8, Kouto 1−1−1, Sayo, Sayo−gun, Hyogo 679−5198, Japan.

[4]Synchrotron X−ray Station at SPring−8, National Institute for Materials Science, Kouto 1−1−1, Sayo−gun, Hyogo 679−5148, Japan.

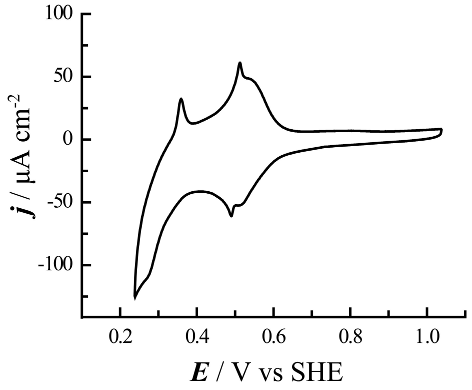

Figure S1. Voltammogram of quasi-Au(111) film on Si prism in 0.5 M H_2SO_4 + 1 mM $CuSO_4$.

Scanning rate is 0.050 V sec^{-1}.

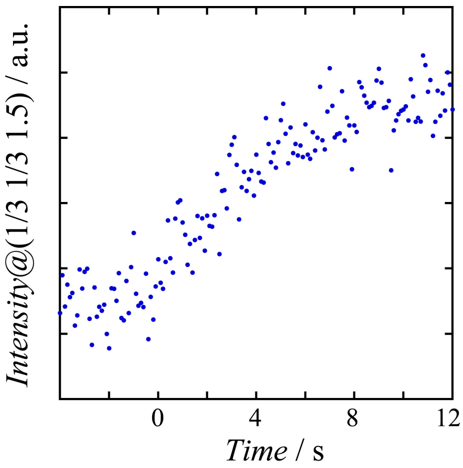

Figure S2. Transient diffraction intensity at the peak position of the (1/3 1/3 1.5) originated form √3 × √3 honeycomb structure. Potential was stepped from 1.05 V to 0.45 V.

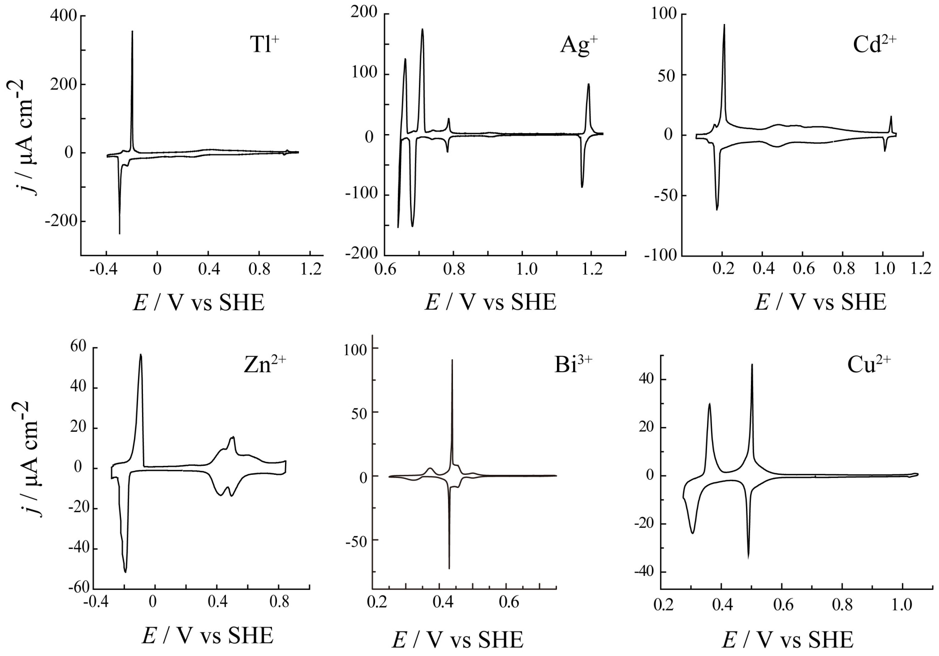

Figure S3. Voltammograms of Au(111) electrode. Tl upd: in 0.5 M H_2SO_4 + 0.5 mM Tl_2SO_4 at scanning rate of 0.050 V sec^{-1}, Ag upd: in 0.5 M H_2SO_4 + 0.5 mM Ag_2SO_4 at scanning rate of 0.010 V sec^{-1}, Cd upd: in 0.5 M H_2SO_4 + 1.0 mM $CdSO_4$ at scanning rate of 0.050 V sec^{-1}, Zn upd: in 0.5 M Na_2HPO_4 + 1.0 mM $Zn(ClO_4)_2$ at scanning rate of 0.050 V sec^{-1}, Bi upd: 1.0 M $HClO_4$ + 0.5 mM Bi_2O_3 at scanning rate of 0.002 V sec^{-1}, Cu upd: in 0.5 M H_2SO_4 + 1.0 mM $CuSO_4$ at scanning rate of 0.005 V sec^{-1},

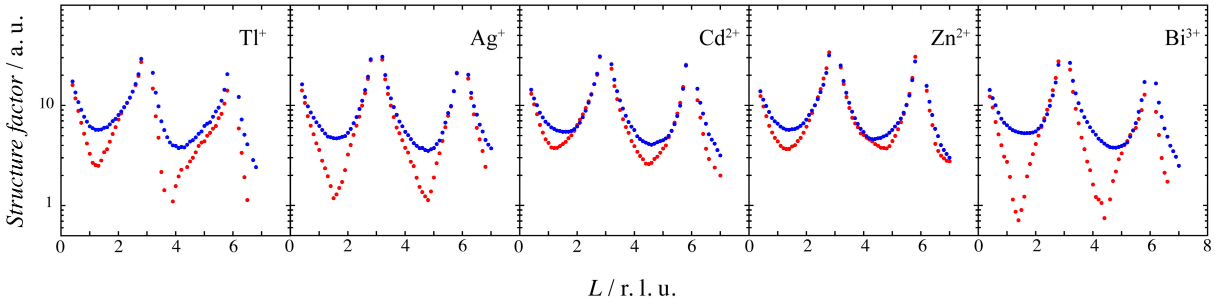

Figure S4. Specular crystal truncation rods of Au(111) electrode. Tl upd: in 0.5 M H_2SO_4 + 0.5 mM Tl_2SO_4 at 0.20 V(blue) and -0.40 V (red), Ag upd: in 0.5 M H_2SO_4 + 0.5 mM at 1.25 V (blue) and 0.75 V (red), Cd upd: in 0.5 M H_2SO_4 + 1.0 mM $CdSO_4$ at 0.60 V(blue) and 0.10 V (red), Zn upd: in 0.5 M

Na_2HPO_4 + 1.0 mM $Zn(ClO_4)_2$ at 0.30 V(blue) and -0.30 V (red), Bi upd: 1.0 M $HClO_4$ + 0.5 mM Bi_2O_3 at 0.97 V(blue) and 0.37 V (red),
